# The Type I+ Forehead in Facial Feminization Surgery

**DOI:** 10.1007/s00266-024-04341-2

**Published:** 2024-09-18

**Authors:** Shahrzad Moghadam, Kaavian Shariati, Kelly X. Huang, Madeline G. Chin, Jonnby S. LaGuardia, Meiwand Bedar, Sumun Khetpal, Brendan J. Cronin, Justine C. Lee

**Affiliations:** 1 Division of Plastic and Reconstructive Surgery, David Geffen School of Medicine, University of California, 200 Medical Plaza Suite 460, Los Angeles, CA 90095-6960 USA; 2 David Geffen School of Medicine, UCLA Gender Health Program, University of California, Los Angeles, Los Angeles, CA 90095 USA

**Keywords:** Facial feminization surgery, Feminizing forehead reconstruction, Gender-affirming facial surgery, Facial gender-affirming surgery

## Abstract

**Background:**

Feminizing fronto-orbital reconstruction involves one of four possibilities with the Ousterhout Type III anterior table frontal sinus osteotomy and setback performed in most patients while the Type I reduction recontouring is reserved for patients without frontal sinuses or thick anterior tables. However, patients with frontal sinuses and either a moderately thick anterior table or a shallow frontal sinus in the sagittal plane represent an intermediate morphology. For such morphologies, we introduce the novel Type I+ fronto-orbital reconstruction technique, consisting of frontal bone recontouring supplemented with anterior table reconstruction and split cranial bone graft.

**Methods:**

Transgender and gender non-conforming patients who underwent Type I+ or Type III feminizing fronto-orbital reconstruction (2019–2023) were included for retrospective review and comparison of techniques.

**Results:**

In the 123 patients (mean age 32.2 ± 9.5 years) included, 6.5% underwent Type I+ and 94.5% underwent Type III feminizing fronto-orbital reconstruction. Morphologically, Type I+ patients displayed a shallower frontal sinus compared to Type III patients (median anterior to posterior table depth 4.1[interquartile range, IQR, 1.1-5.0] versus 9.8[IQR 7.5-12.0]mm, *p*<0.001). At the maximum prominence, Type I+ patients also demonstrated thicker anterior tables compared to Type III patients (median 6.6[IQR 5.0-8.8] versus 2.2[IQR 0.4-4.7]mm, *p*=0.001). Patients receiving Type I+ procedures underwent an anterior table reduction of 2.7±1.2mm versus 4.2 ± 1.2mm for Type III procedures in the sagittal plane (*p*=0.002).

**Conclusions:**

The current work introduces a novel solution to an intermediate frontal sinus phenotype for gender-affirming facial feminization surgery.

**Level of Evidence IV:**

This journal requires that authors assign a level of evidence to each article. For a full description of these Evidence-Based Medicine ratings, please refer to the Table of Contents or the online Instructions to Authors www.springer.com/00266.

## Introduction

In the past decade, an increase in US health insurance coverage for facial gender-affirming surgeries has translated to a rapid increase in surgical experience. Feminizing fronto-orbital reconstruction, traditionally based on the Ousterhout forehead classification, is one of the most powerful and important procedures for patients.[3–7] Within Ousterhout’s system, the Type III forehead, the most common phenotype, describes bossing secondary to positioning and curvature of the anterior table of the frontal sinus. Thus, the most common skeletal forehead feminizing procedure is the anterior table setback. The Type I forehead, the second most common phenotype treated by recontouring alone, describes bossing in patients born without frontal sinuses or with a thick anterior table such that the frontal sinus is posterior to the area responsible for bossing. Type II and IV foreheads occur less frequently and involve partial reduction with augmentation camouflage in the former or augmentation alone in the latter. Although the Ousterhout classification has provided a useful framework for many surgeons, intermediate scenarios have emerged. 

**Fig. 1:**
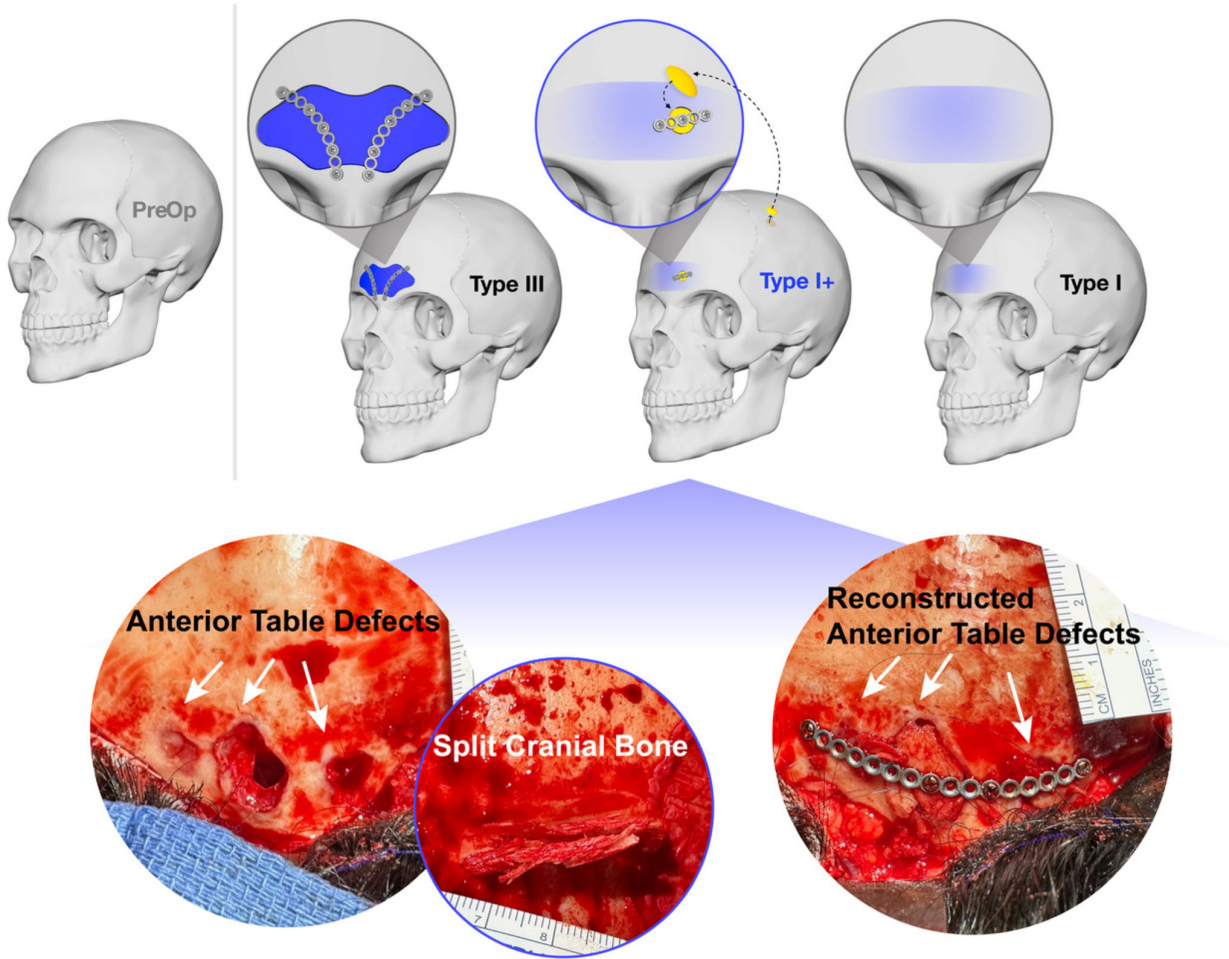
Schematic demonstrating forehead feminization through Type III, Type I+, and Type I reduction methods in frontal and left oblique views. Dark blue, light blue, and yellow represent osteotomized frontal sinus setback, split cranial bone grafting, and anterior table recontouring, respectively. Intraoperative Type I+ reduction photographs, from left to right, depict anterior table defects, outer table split parietal cranial bone graft harvest, and bone graft stabilization with titanium plates and screws. Copyrights retained by Justine C. Lee

Occasionally, patients present with a frontal sinus configuration where a Type III setback would be excessive, yet a Type I approach would be insufficient. In such cases, we introduce a novel “Type I+” feminizing fronto-orbital reconstruction technique involving frontal bone recontouring combined with split cranial bone grafting for reconstruction of anterior table defects (Fig. 1).

## Methods

### Patients

Transgender and gender non-conforming patients assigned male at birth who completed primary facial feminization surgery by a single surgeon (2019–2023) were retrospectively reviewed (IRB#19-001482). Patients with a frontal sinus who underwent either Type I+ or Type III fronto-orbital reconstructions were included and compared using independent samples Mann–Whitney U tests or Fisher’s exact tests (SPSS Version 28, Chicago, IL). Patients without frontal sinuses were excluded.

### Type I+ Patient Selection and Surgical Technique

**Fig. 2:**
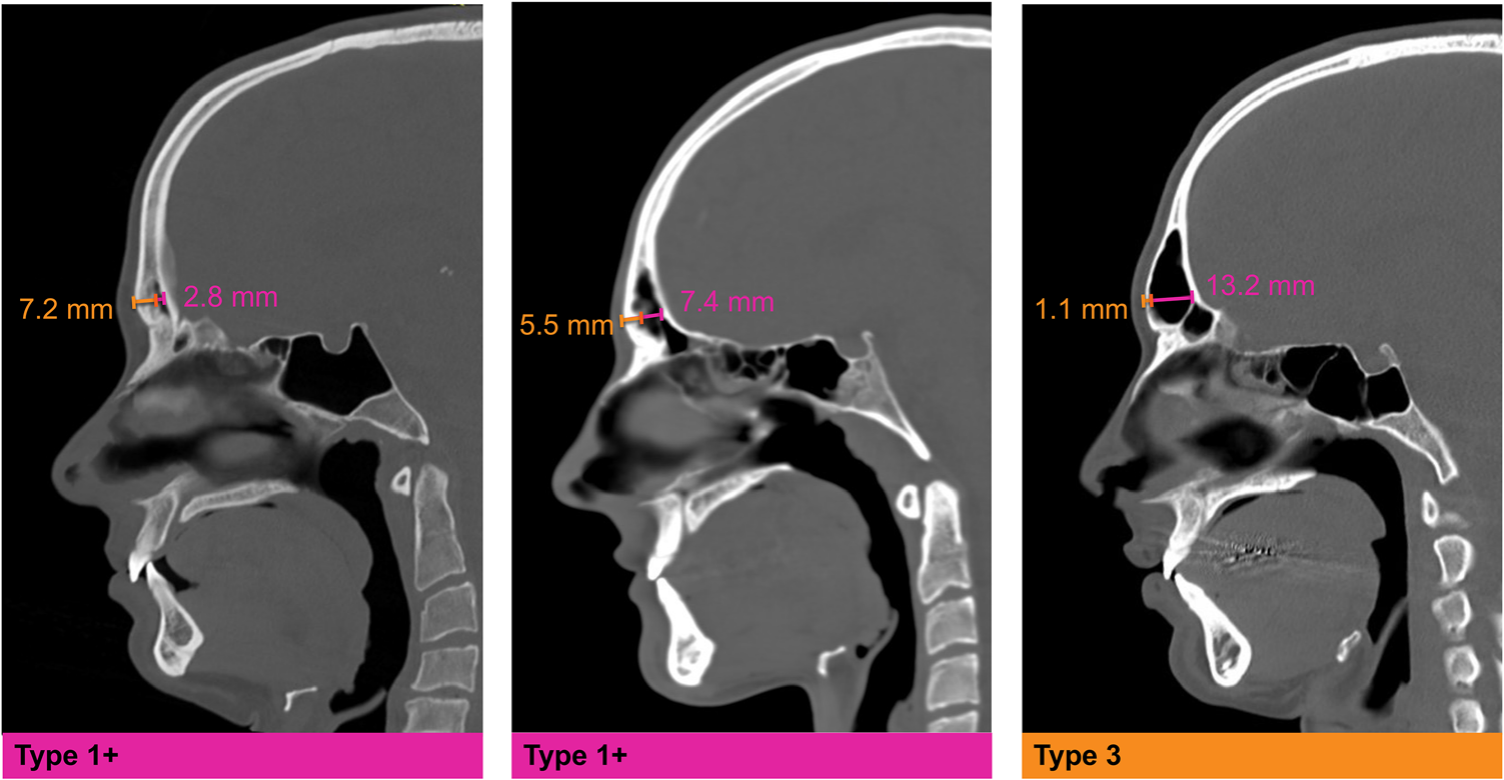
Preoperative sagittal craniofacial computerized tomography images of patients who underwent a Type I+ versus a Type III forehead reduction. On the left, patients with a thick anterior table (5.5 mm) and shallow frontal sinus (7.4 mm) are candidates for a Type I+ reduction. Depicted on the right, patients with a Type III forehead presenting with a thinner anterior table (1.1 mm) and a greater frontal sinus depth (13.2 mm) require a setback procedure. Copyrights retained by Justine C. Lee

Clinical exam findings and virtual modeling using preoperative, fine-cut computed tomographic scans as we have previously described [8, 9] were used to determine the quantity of forehead reduction necessary for feminization. Selection for Type I+ fronto-orbital reconstruction was based on the following criteria: 1. Narrow frontal sinus such that a Type III anterior table setback would result in an excessively narrow sinus (<5 mm) in the sagittal plane. 2. A thick enough anterior table such that Type I recontouring is sufficient for the majority of the bone with small areas of full-thickness defects of the anterior table (Fig. 2).

For the Type I+ forehead, the porcupine frontal bone recontouring guide that we previously described [8, 9] is used in a manner identical to that used for a standard Type I forehead or Type III forehead. The major difference in the Type I+ technique is that there is an intentional entry into the frontal sinus with the creation of a full-thickness anterior table defect in the specified areas, which is then reconstructed using split cranial bone graft. Defects typically range in number and size, ranging up to 1 x 2 cm in area. For the Type III forehead, a combination of a frontal sinus guide and a porcupine frontal bone recontouring guide is used.[8, 9] Following osteotomy of the frontal bone, the frontal bone is recontoured and the anterior table is then setback and secured with titanium plates and screws.

## Results

In the 123 patients (mean age of 32.2±9.5 years) included, 8 patients (6.5%) underwent a Type I+ and 115 patients (93.5%) underwent Type III fronto-orbital reconstruction **(**Table 1**)**. Anatomically, patients who underwent Type I+ procedures displayed a shallower frontal sinus compared to those who received Type III procedures (median maximum depth of 4.1[interquartile range, IQR 1.1-5.0] versus 9.8[IQR 7.5-12.0] mm, *p*<0.001). The anterior table thickness was also 3.0-fold greater in Type I+ patients. Consistent with the idea of an intermediate phenotype, Type I+ patients underwent less reduction of the anterior table compared to Type III patients (2.7±1.2 vs. 4.2±1.2 mm, *p*=0.002). No complications were noted in the Type I+ patients while one Type III patient developed a mucocele postoperatively and was treated with re-advancement of the anterior table.

**Table 1:** Patient and surgical characteristics

	Fronto-Orbital Reconstructive Procedure Type			
	Total Cohort	Type I+	Type III	*p value*
	n=123	n=8	n=115	
Age at surgery, years, mean (SD)^a^	32.2 (9.5)	30.9 (9.0)	32.3 (9.5)	0.75
BMI at surgery, kg/m^2^, mean (SD)^a^	25.0 (5.3)	23.3 (2.9)	25.1 (5.4)	0.49
*Gender type, n(%)*^*b*^				
Binary	109 (88.6)	6 (75.0)	103 (89.6)	0.23
Nonbinary	14 (11.4)	2 (25.0)	12 (10.4)	
*Race/ethnicity, n(%)*^*b*^				
Asian	13 (10.6)	2 (25.0)	11 (9.6)	0.67
Black	5 (4.1)	0 (0.0)	5 (4.3)	
Caucasian	70 (56.8)	5 (62.5)	65 (56.5)	
Latinx	30 (24.4)	1 (12.5)	29 (25.3)	
Other	5 (4.1)	0(0.0)	5 (4.3)	
Hormone therapy duration, years, median(IQR)^a^	3.4 (2.2-5.5)	2.6 (1.7-6.6)	4.9 (2.2-5.5)	0.42
Anterior *to posterior table depth at maximal prominence, mm,* median (IQR)^a^	9.6 (7.1-11.8)	4.1 (1.1-5.0)	9.8 (7.5-12.0)	**<0.001**
Anterior table thickness at maximal prominence, mm, median (IQR)^a^	3.3 (0.5-5.0)	6.6 (5.0-8.8)	2.2 (0.4-4.7)	**0.001**
Anterior table reduction at maximal prominence in the sagittal plane, mm, mean (SD)^a^	4.0 (1.3)	2.7 (1.2)	4.2 (1.2)	**0.002**
*Simultaneous soft tissue forehead procedures performed, n(%)*^*b*^				
Hairline advancement	82 (66.7)	6 (75.0)	76 (66.1)	0.72
Browlift	121 (98.4)	7 (87.5)	114 (99.1)	0.17
Duration follow-up, months, median (IQR)^a^	6.0 (1.0-12.0)	2.5 (0.0-10.3)	6.0 (1.0-12.0)	0.17
Forehead complications, n(%)^b,c^	1 (0.8)	0 (0.0)	1 (0.9)	1.0

^a^ Independent samples Mann–Whitney U test

^b^ Fisher’s exact test

^c^ Consisted of one mucocele

*BMI;* Body mass index

## Discussion

**Fig. 3:**
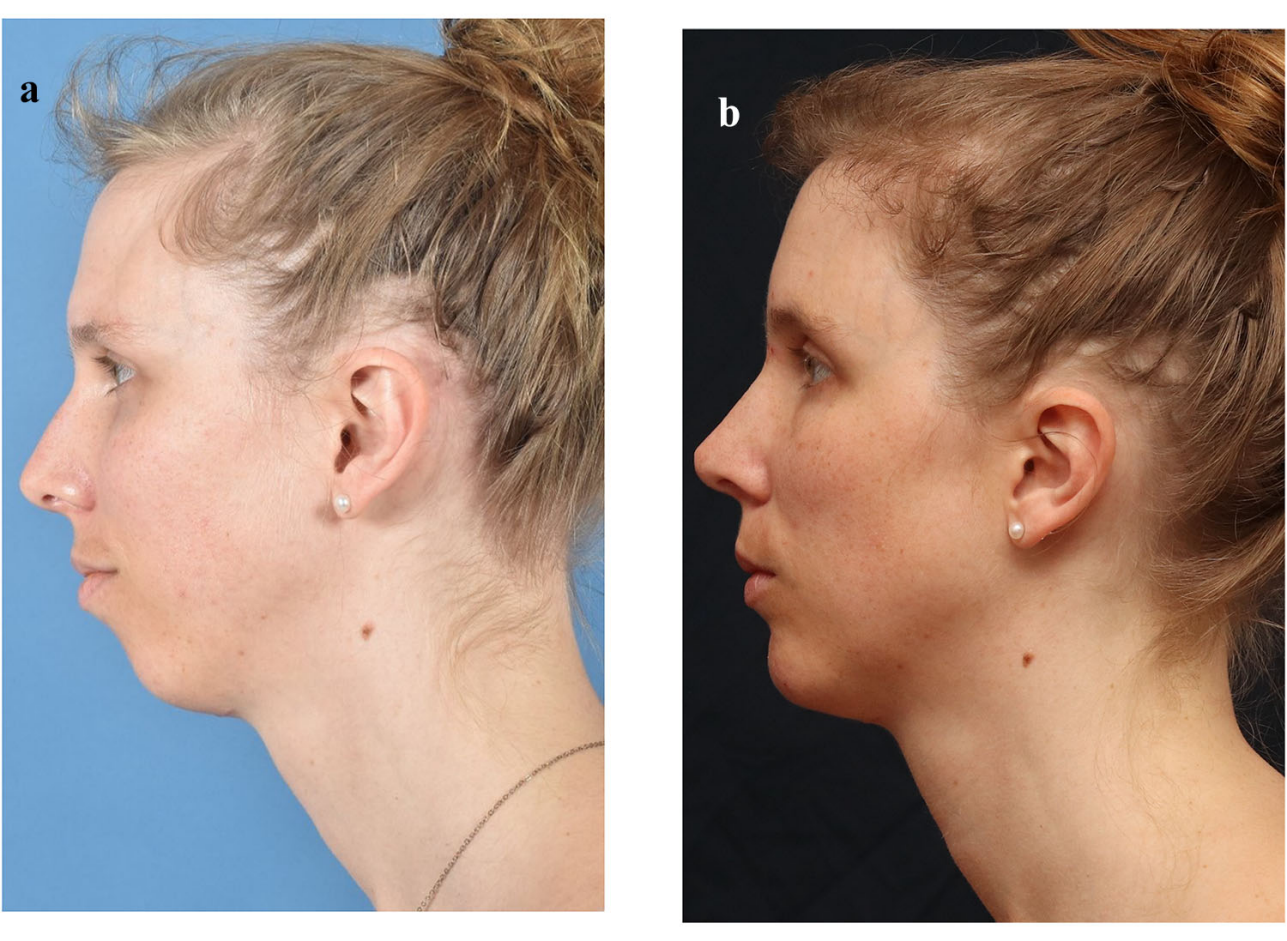
Left lateral views of preoperative (**a**) and 8-month postoperative (**b)** photographs of a patient following Type I+ forehead reduction during facial feminization surgery. The rationale for a Type I+ reduction for this patient was due to the presence of a shallow sinus between the anterior and posterior tables preoperatively. The patient also received hairline reduction, brow lift, septorhinoplasty, osseous sliding genioplasty, and bilateral gonial angle reduction. Copyrights retained by Justine C. Lee

The introduction of modern craniofacial techniques in imaging and planning for facial gender-affirming surgery has resolved intermediate phenotypes that do not fit classically into the methods previously described by Ousterhout. We have identified two such instances appropriate for an alternative intermediate surgical solution that we have termed Type I+ **(**Fig. 3**)**.

The rationale for a Type I+ approach was born out of two observations. First, while rare, we have encountered a mucocele in our Type III reconstructions. Unlike mucoceles found in traumatic frontal sinus fractures, the nasofrontal duct was patent and the presence of the mucocele was due to fusion of the anterior and posterior tables superior to the nasofrontal duct, concluding that excessive narrowing of the frontal sinus in the sagittal plane had occurred. This patient was successfully treated with frontal bone re-advancement. Currently, it is unclear how much space is necessary to prevent mucoceles following anterior table setback; however, these studies are underway. In such patients with shallow sinuses, the distance between the anterior and posterior tables at the inferior aspect of the frontal sinus is typically still large enough to permit reduction of the anterior table. To prevent any narrowing superiorly, we suggest that the Type I+ procedure is potentially safer than Type III, while allowing for more reduction than the Type I procedure. The second observation was that some patients were on the verge between a Type I and Type III procedure as a sinus was present but the anterior table was mostly thick. In such cases, the decision-making process was whether the osteotomy of the anterior table would result in a larger defect or if the defect generated at areas of maximum prominence after burring would be larger. If burring generated smaller anterior table defects, we elected to use the Type I+ procedure.

The primary limitation in this study is that it is a description of a novel technique applicable to a small percentage of patients. Hence, future multi-year follow-ups to assess the sequelae of these procedures will be valuable to understand long-term outcomes.
